# Transactions of the Buffalo Medical Association, February 1st, 1876

**Published:** 1876-04

**Authors:** 


ART. IV.—*Buffalo Medical Association.* Tuesday Evening, February 1st, 1876.

The Vice-President, Dr. Wyckoff, in the chair.

Members pressent: Drs. White, Wyckoff, Cronyn, Willoughby, O'Brien, Brecht, Mynter, Little, Bartlett, Boysen, Hopkins, Fowler, Briggs, Thos. Lothrop, Folwell, Trowbridge, Harding, Howe,

Samo, Wetmore, Coxe, T. M. Johnson and Southwick. Dr. Shannon and Pettit by invitation.

Minutes read and approved.

Dr. Willoughby was appointed as essayist for the next meeting.

Dr. Mynter, under voluntary communications, read a paper on the operative treatment of pleuritic effusions, and exhibited the apparatus which he used in aspirating the pleural cavity.

Dr. Wetmore, the essayist for the evening, read a paper on the Sympathetic Nervous Syssem. See page 328.

Dr. White said that he came as a learner and had not been disappointed. The Dr. referred to having witnessed the experiments of Bernard upon the sympathetic system, after witnessing these he went to London, where he fell in with Robert Lee, who had recently been making dissections of Uteri, both of the virgin and in those who had been pregnant. Lee was kind enough to show him these dissections and demonstrations. He said that Dr. Wetmore had spoken of nausea and vomiting supervening upon pregnancy, and if he understood rightly, he had said it was due to a peculiar condition of the mucous membrane. He could not agree with this, he agreed that it was occasioned through the sympathetic nerve.

Moreau attributes it to the compression of the Uterus, it having descended and being confined to the limits of the pelvis.

In several instances which had occured in his practice, where retroversion or retroflexion had coexisted with pregnancy, the nausea had been greatly increased. There is no point in the sympathetic nervous system which is so interesting as its relation to the uterine system.

He referred to the case of a young lady, who consulted him for loss of voice, which had been of some weeks duration. The patient was not hysterical, and had never had a similar attack. Laryngoscopic examination made by Dr. Brush revealed a partial paralysis of the vocal cords. No local condition could be found as the cause. A careful inquiry revealed the fact that the patient was suffering from Uterine disease. It being treated and cured, the voice was restored.

He had under treatment another case, on two different occassions, for a similar conditions of things. The patient was the wife and daughter of a physician. The loss of voice, which occured first ten years ago, and again within a few months, was due to retroflexion. Neither the father, or husband, nor himself were inclined to attribute the loss of voice to hysteria. In conclusion, he expressed his thanks to Dr. Wetmore for his excellent paper.

Dr. Cronyn said if he were to have suggested a theme for the evening he would have asked him to enlarge upon this very subject. His only fault was that it was too short. In 1850 and '52, Dr. Davis, a gentleman in one of the provincial towns in England, was inclined to lay great stress upon the importance of the function exercised by the solar plexus. At that time, 1851-52, the speaker had a patient who was suffering with obstinate nausea. Dr. Flint, then residing in Buffalo, saw the patient in consultation, but could suggest no relief. The patient retained nothing upon the stomach, and was only sustained by means of enema. Reading Dr. Davis' lecture, he put some of his ideas into practice and applied a blister opposite the solar plexus, and in a few hours the patient was able to retain food.

We find serous effusion, arrested by galvanism and other means, which influencing the organs primarily engaged, cures the effusion.

With regard to Robert Lee's experiments, he had no doubt as to their truth and exactness.

It will be found everywhere in the body, that the ganglionic system has a controlling influence. A summary might be made that they are the life of the body.

Dr. Bartlett would thank Dr. Wetmore. He said that the more we desire to advance in medical science, the more we must study the nervous system. He wished also to thank Dr. Mynter; thought that both papers were a credit to the society.

Dr. Howe read the following :

"In a paper which I had the honor of presenting at a former meeting of this society, I mentioned the fact that irritating gasses and vapors often exert an injurious effect upon the eye. This called forth from one of the members some statements in regard to the injurious results from a similar cause experienced by the workmen in the tunnel at the water works, then being constructed under the Niagara River. The few facts given at that time were so entirely new to the most of those present, and so interesting to myself, that I determined to give the subject the study which its importance in an ophthalmic point of view demanded. A brief resumé of the conclusions arrived at, may seem worthy of attention, if only as a matter of local interest.

In order to show the relative position of the various parts of the tunnel, I present here a diagram illustrating the general plan of its construction. The shaft at the shore end is 67 feet deep, 310 feet out, there is a second shaft 57 feet deep sunk from Bird Island Pier. The shaft at the inlet pier is 40 feet deep, and the entire tunnel, which is 1020 feet long, has an inclination upwards of 13 feet.

The work was begun on the 12th of May, 1870, and advanced without any special difficulty until the first shaft had been sunk to the depth of 47 feet. Thus far it was necessary to cut through layers of solid rock, but here a stratum of loose, pliable limestone was struck, some 6 to 8 inches thick. It was through this that the sulphurous gas permeated. Immediately, upon reaching that point, the workmen began to have trouble with their eyes, which became swollen and tender, and the longer they were exposed the worse the inflammation grew. There was one time when excavating this portion that every man was effected to a greater or less extent. This point being passed, the inconvenience was temporarily lessened. Soon it was manifested again however, when the same stratum of rock was cut by the sinking of the second shaft, where the trouble was experienced, and in an equal degree. When the excavation was advanced about 500 feet beyond this middle pier, work was temporarily suspended. During that time the gas had an opportunity to collect in such quantities that upon resuming operations, two men who entered the Bird Island shaft, were taken up, a short time after descending, in a practically blinded condition. From this second point until near the end of the tunnel no unpleasant effect were experienced. At the latter part, however, the same stratum was met with a third time, and would have proved troublesome, but for the perfection to which the ventilation had been carried by means of condensed air. Such is the account of the work as given by the chief engineer, Mr. Knapp, who kindly furnished me most of the data.

The general form of the disease thus produced, was that of a conjunctivitis. Not being in Buffalo at the time when these cases were most frequent, my personal observations did not begin till a later date, but I have been assured by physicians that such was its character, and the statements of the workmen as well as the result of the treatment, confirmed that diagnosis. The inflammation would show itself in a time varying from an hour to a day and attain a condition which ranged from the slight catarrhal form to the most violent purulent type. Removal from the irritating causes, cooling applications, or the workmen's favorite remedy, a potato poultice, usually produced a cure in a few days. Some were incapacitated for work for a week or more, but in general the worst was over in three or four days. The principal opportunity I have had of studying this process has been upon my own eyes. Finding that they were particularly susceptible to the influence, I could notice not only the different changes in the course of the disease, but what remedy produced the best results, and I became convinced that it was a conjunctivitis to be treated in the usual manner.

Seeing thus the effects which are produced by the gas, we naturally inquire more exactly as to its character. I have spoken of it as sulphurous, and so it is. Immediately on descending the shaft, the peculiar odor of rotten eggs that greets ones nostrils leaves but little doubt as to the presence of sulphuretted Hydrogen. At several places there could be seen veins through which water came, having a distinct sulphurous taste, while the rocks in that vicinity were covered with a whitish slime. For the purpose of analysis I brought away specimens or both the water and the deposit. The former soon lost its peculiarity from exposure to the air, while the deposit, which was white, changed its color by decomposition but still retains its original perfume. To ascertain its character beyond doubt an analysis was made by a professional chemist, Prof. W. H. Pitt, and the result showed that the conjectures were right. The presence of sulphur in such localities is nothing unusual. It occurs in another part of this city, and almost everywhere in rocks when there is that process going on in the vicinity, which is known as destructive distillation. The former forests of the carboniferous age have been converted into coal in some places, and in others into petroleum, carburetted Hydrogen (marsh gas), or into sulphurretted Hydrogon. In this tunnel, then, the workmen simply struck sulphurretted Hydrogen instead of striking oil.

The question of inetrest to medical men is not how it came there, but how it produced these effects on the workmen's eyes. This is a matter upon which a very little chemistry throws a great deal of light.

An analysis of the tears shows the solid portion of that secretion to consist almost entirely, of common salt, Sodium Chloride. Now it is found by chemists that Sulphur has a great affinity for Sodium, and that Hydrogen unites readily with Chlorine. This reaction occurring on the eye there results Sodium Sulphide and Hydrochloric Acid, two extremely irritating substances. To illustrate this effect, I have subjected the eyes of a rabbit to the influence of these substances in the proportions in which they combine, and as a result the conjunctiva is seen to be swollen and inflamed in a marked degree.

Such a chemical change, in general terms, is a rational, and to me the only manner of accounting for the symptoms. Knowing therefore, what the substance is and how it acts, there remains only the therapeutical question of how the unpleasant effects could be obviated in future cases. This is something not so easily done. Theoretically, the best way would be to apply to the eye some substance having a greater affinity for sulphur than has sodium. But practically, there is the objection that any such remedy in such a position would be almost as bad as the disease. It occured to me, therefore, to place this third substance, not on, but near the eye, and for that purpose constructed the spectacles which I have here. They consist of an ordinary pair of gauze protectors or goggles, having the wire gauze covered with a piece of cloth. This cloth is intended to be wet with a saturated solution of Acetate of Lead—a fluid which combines readily with sulphurretted Hydrogen—and so to speak absorbs the gas in its vicinity. In order to test their value I covered one eye in this way and entered the tunnel, remaining in the parts where the gas was the most dense for an hour or more. At the end of that time the unprotected eye was sensibly effected, and the other was perfectly normal, while the cloth had become blackened by a deposit of the Sulphide of Lead.

For workmen in such situations I must say howerer, that anything of this sort is open to the objection that after a time it is rendered useless by the condensation of the moisture within. That the contrivance falls short of perfectly fulfilling the object, I know, but it is, at least, of temporiary benefit, and is superior to anything thus far proposed.

Dr. Bartlett said that if the sulphur in the tunnel has been so prominent as to have such an effect on eyes the water coming through would not be pure.

Dr. Hopkins read an article in reference to the sanitary authorities of the city, and introduced the following resolution:

*Resolved*, That a committee of three, to be known as the sanitary committee, be appointed to investigate the regulations of our health department, and all matters pertaining to the sanitary government of our city, and to report to this Association at its next annnal meeting, with such recommendations as they may deem proper, in order that this Association may appreciate, as fully as may be, the entire sanitary government of the city of Buffalo.

Dr. White said that he merely wished to assert a firm protest against Dr. Hopkins' assumption that the society had made no action in regard to public health. No longer ago than the last epidemic of scarlet fever the society had met and given publicity to its views on the subject.

The Chair appointed Drs. Hopkins, Folwell and Barnes as the committee.

Dr. White moved that the paper of Dr. Mynter lie upon the table until next meeting. Carried.

On motion the Association adjourned.

